# The application of Signalling Theory to health-related trust problems: The example of herbal clinics in Ghana and Tanzania

**DOI:** 10.1016/j.socscimed.2017.07.009

**Published:** 2017-09

**Authors:** Kate Hampshire, Heather Hamill, Simon Mariwah, Joseph Mwanga, Daniel Amoako-Sakyi

**Affiliations:** a Dept of Anthropology, Durham University, UK; b Dept of Sociology, Oxford University, UK; c Dept of Geography and Regional Planning, University of Cape Coast, Ghana; d National Institute for Medical Research, Tanzania; e School for Medicine, University of Cape Coast, Ghana

**Keywords:** Africa, Behavioural Game Theory, Uncertainty, Herbal medicine, Traditional medicine, Health-seeking behaviour, Decision-making, Qualitative research

## Abstract

In contexts where healthcare regulation is weak and levels of uncertainty high, how do patients decide whom and what to trust? In this paper, we explore the potential for using Signalling Theory (ST, a form of Behavioural Game Theory) to investigate health-related trust problems under conditions of uncertainty, using the empirical example of ‘herbal clinics’ in Ghana and Tanzania. Qualitative, ethnographic fieldwork was conducted over an eight-month period (2015–2016) in eight herbal clinics in Ghana and ten in Tanzania, including semi-structured interviews with herbalists (N = 18) and patients (N = 68), plus detailed ethnographic observations and twenty additional key informant interviews. The data were used to explore four ST-derived predictions, relating to herbalists' strategic communication (‘signalling’) of their trustworthiness to patients, and patients' interpretation of those signals. Signalling Theory is shown to provide a useful analytical framework, allowing us to go beyond the *primary trust problem* addressed by other researchers – cataloguing observable indicators of trustworthiness – and providing tools for tackling the trickier *secondary trust problem*, where the trustworthiness of those indicators must be ascertained. Signalling Theory also enables a basis for comparative work between different empirical contexts that share the underlying condition of uncertainty.

## Highlights

• Applies Signalling Theory (ST) to patient-herbalist trust in Ghana & Tanzania.
• Herbalists communicate trustworthiness to patients via observable ‘signals’.
• Patients interpret signal reliability based on perceived costs and pay-offs.
• In conditions of uncertainty, informational mismatches result in misplaced trust.
• ST allows us to go beyond description, enabling prediction and comparative work.

## 1 Introduction

### 1.1 Healthcare and the problem of trust

Across low/middle-income countries (LMICs), a combination of weak health systems and high demand leads many people to resort to a poorly-regulated ‘informal’ sector with substantial uncertainty about the quality of treatments on offer. Patients are thus faced with the problem of whom and what to *trust*. Trust usually implies *vulnerability*, since the truster has to depend on, but can never be certain about, another's motives, intentions and future actions (Baier, 1994, Gilson et al., 2005). With healthcare, the stakes are particularly high: individuals' health can be severely compromised by taking harmful products, while poor quality or ineffective treatment can be equally dangerous when it delays effective care and/or undermines trust in healthcare more generally (Blair et al., 2017).

The role of trust in healthcare has received significant research attention in recent years. Most work has focussed on doctor-patient relationships in ‘Western’ settings (e.g. Brown, 2009, Calnan and Rowe, 2008, Barrett et al., 2007, Mechanic and Meyer, 2000, Meyer, 2015), but research has increasingly included resource-poor contexts, where inadequate service provision and financial barriers may severely constrain choice (e.g. Birungi, 1998, Gilson et al., 2005, Russell, 2005, Ozawa and Walker, 2011, Tibandebage and Mackintosh, 2005, Ackatia-Armah et al., 2016, Rodriguez, 2016). These studies have identified various inter-personal factors (honesty, sincerity, empathy, evidence of competence, etc.) and institutional factors (trust in medical training, general trust in public institutions, etc.) that interact to promote trust and influence treatment-seeking decisions.

However, this literature suffers two important limitations. First, it assumes that it is *intrinsically* a good thing for patients to trust practitioners, as the basis for effective care. Thus, papers often conclude with recommendations that health professionals hone their listening/communication skills, etc. to foster patient trust (e.g. Ackatia-Armah et al., 2016, Gilson, 2003, Mechanic and Meyer, 2000). This is fine if the practitioner really *is* trustworthy but, in the highly unregulated informal sector present in many LMICs, this cannot necessarily be assumed. If behaviours that engender trust can be taught and learned, it follows that they can also be *mimicked*. This leads to the second shortcoming: current literature tends to be limited to *describing* and *classifying* qualities associated with trustworthiness (competence, integrity, empathy, etc.) and, sometimes, the observable indicators of those qualities (making eye contact, smiling, listening, etc.). However, given the risk of fakery, how can patients determine which indicators can be trusted?

In this paper, we propose that **Signalling Theory** – a variant of Behavioural Game Theory – might provide a valuable tool for enabling a deeper and more theoretically-informed analysis of health-related trust problems. Signalling Theory, whose origins lie in economics (Akerlof, 1970, Spence, 1973: Podolny, 2005) and evolutionary biology (Zahavi and Zahavi, 1997), has more recently been used by biological anthropologists (Bliege Bird and Smith, 2005, Sosis and Alcorta, 2003) and sociologists (Bacharach and Gambetta, 2003, Gambetta and Hamill, 2005, Gambetta, 2009, Hamill, 2011) to understand how communication works under conditions of uncertainty. However, Game Theory has rarely been applied to healthcare (see Tarrant et al., 2010; for a notable exception) and, to our knowledge, Signalling Theory has never been used for this purpose. Below, we outline, and then apply, the principles of Signalling Theory to a scenario where uncertainty is particularly high: ‘herbal clinics’ in Ghana and Tanzania. Our aim is twofold: to address an empirical question – how, under conditions of uncertainty and informational asymmetry, patients come to trust/distrust herbalists and their medicines – and to assess the potential contribution of Signalling Theory to the study of health-related trust problems more widely.

We use the terms ‘*trust*’ and ‘*trustworthiness*’ here in a very specific way. When we say that a patient ‘trusts’ a practitioner or medicine, we mean that they trust *enough* to accept a specific treatment at a particular moment; not necessarily that they trust the practitioner/medicine more generally. Neither do we assume that trust is the *only* factor driving treatment-seeking decisions, especially in populations facing serious resource constraints, as we discuss below.

### 1.2 Signalling Theory

Signalling theory (ST) addresses the problem of how individuals communicate unobservable properties like trustworthiness to one another in contexts of uncertainty and asymmetrical information. Because trustworthiness (like honesty or courage) cannot be directly observed, we have to discern it through associated behaviour or ‘signals’. The **primary trust problem** is to ascertain an individual's trustworthiness by looking for observable indicators of that ‘property’. However, as noted above, some people – ‘mimics’ – may display the same signals, in order to dupe someone else to their advantage. The **secondary trust problem** is thus to determine whether the signals of trustworthiness can themselves be trusted.

To distinguish between genuinely trustworthy individuals and ‘mimics’, the receiver (the one ‘reading’ the signals) must try to evaluate the cost of signal production (resources, time, etc.) relative to expected pay-offs for the signaller. ST distinguishes three categories of signals according to their discriminatory power. **Pooling signals** can be displayed easily and cheaply by genuine and dishonest signallers alike (for example, smiling) so cannot distinguish effectively between the two. **Semi-sorting signals** carry greater costs for dishonest signallers, who are therefore less likely to display them than honest ones. Semi-sorting signals thus convey more information about trustworthiness, although they may still be faked by an imposter who anticipates a sufficient pay-off to justify the investment. Fully **discriminating signals** distinguish reliably between honest and dishonest signallers because they would be beyond the latter's capacity to mimic, given the expected pay-off (Spence, 1973, Gambetta, 2009). Gambetta and Hamill (2005) have noted that, in ‘real life’, the situation is usually better represented by *continuum* of signals that convey varying degrees of imperfect information, operating both singly and in clusters.

Although ST predicts that signalling strategies everywhere draw on a similar underlying logic, the signals themselves will be *context-specific*. A behaviour that signals trustworthiness in one context may signify something different (or nothing at all) in another. A signal's discriminatory power also varies over *time*, as once-discriminating practices become easier to mimic and are more widely adopted. Actors therefore need relevant, up-to-date knowledge regarding the costs and pay-offs of signals used in a particular context (Gambetta and Hamill, 2005:14). Trustworthiness is also *situation*-specific: you may trust a healer to prescribe appropriate medicine but not to look after your children, or to treat a headache but not cancer.

### 1.3 Applying Signalling Theory to the case of ‘herbal clinics’ in Ghana and Tanzania

Herbal clinics have become an increasingly prominent feature of Africa's therapeutic landscapes, especially in urban areas. Operated usually by men, they claim to combine the best of ‘indigenous’ healing with ‘modern’, ‘scientific’ approaches, positioning themselves strategically within highly-competitive markets. Herbal clinics in contemporary Ghana and Tanzania range from large, up-market, ‘high-tech’ establishments to basic, single-room structures (see Marsland, 2007, Hsu, 2002, McMillen, 2004, Langwick, 2010 re Tanzania, and Hampshire and Owusu, 2013; Tsey, 1997, Twumasi and Warren, 1986; re Ghana). Nonetheless, ‘herbal clinics’ share some common features which form the basis of our working definition: a fixed premises; operated by a practitioner claiming expertise in ‘traditional’ herbal medicine; producing and selling manufactured, plant-derived capsules, powders, ointments and/or bottled medicines.

Formal regulation of herbal medicine remains limited in both countries, despite numerous efforts. Since 1992, all herbal products sold in Ghana require approval by the Food and Drugs Authority (FDA), which tests products for acute toxicity (although not efficacy or chronic toxicity, as we discuss below), while practitioners must be licensed by the statutory Traditional Medicine Practice Council. However, off the record, regulators complained that they lack the capacity to operate effectively beyond the capital city; most herbal medicines remain unapproved and inspections are reportedly intermittent. Regulation in Tanzania is, as one Government official put it, “*way behind Ghana.*” The recently-established Traditional and Alternative Health Practice Council recently began the mammoth task of registering all ‘traditional healers’ in the country, while plans to license herbal medicines have yet to be operationalised.

In the absence of effective regulation, objective evidence about the quality and effectiveness of herbal products is limited, but we can infer substantial variation. Recent in-vivo and in-vitro studies in Ghana (e.g. Amoah et al., 2015, Komlanga et al., 2016, Wilmost et al., 2017) and Tanzania (e.g. Nondo et al., 2016) have detected varying levels of antimicrobial activity and efficacy in commonly-marketed herbal medicines. Even less is known about possible harmful effects of herbal medicines, especially chronic toxicity. Under these conditions, we would **predict**, based on Signalling Theory, that:

1) Herbalists operating clinics will use a range of signals to communicate their trustworthiness to patients.
2) The costliness and reliability of signals will vary, from uninformative **pooling** signals (easily displayed by both high- and low-quality herbalists) through **semi-sorting** signals (more costly for lower-quality herbalists given expected pay-offs) to **discriminating** signals (only displayed by high-quality herbalists).
3) Patients will interpret the reliability of signals according to their knowledge/beliefs about their costs (monetary outlays, investment of time, effort, etc.) and pay-offs (principally financial gain) for the herbalist.
4) Over time, some signals will lose their discriminatory power, and new signals will emerge.

## 2 Fieldwork and methods

This study draws on qualitative, ethnographic data: a relatively new departure in ST research (see Sosis and Alcorta, 2003, Gambetta and Hamill, 2005, Gambetta, 2009, Hamill, 2011, Vaughan, 2009), which tends to rely on statistical modelling. We argue that a qualitative, ethnographic approach is essential to understanding the situation-specific nature of signalling practices, where context is crucial and underlying motivations are rarely discernible from standardised research instruments.

The work underpinning this paper began in 2009–10, when KH undertook ethnographic research in four herbal clinics in Cape Coast, an important provincial centre in southern Ghana (Hampshire and Owusu, 2013). This work provided the basis to develop a series of ST-derived predictions, which guided a more formal set of observations and interviews conducted (2015–16) in eight herbal clinics in Cape Coast and ten in Mwanza: a major regional centre in North-West Tanzania. All but one of the herbalists (in Ghana) were men.

In both towns, we (authors plus research assistants) conducted fieldwork in every herbal clinic that we could identify. In each clinic, the researchers spent 2–3 days conducting detailed observations, alongside semi-structured interviews with the herbalist and patients. As each new patient entered, the researcher introduced herself, explained the research protocol, and requested consent to conduct an interview following their consultation with the herbalist. The vast majority of patients (more than 90%) agreed to this and were interviewed in a quiet corner of the clinic. Business was often slow, and the number of patients interviewed per clinic ranges from zero to twelve: altogether, 35 patients were interviewed in Cape Coast and 33 in Mwanza: Table 1.

**Table 1:** Herbal clinic patient sample.

Age group	Ghana		Tanzania		Totals
	Female	Male	Female	Male	
20-39y	10	9	17	6	42
40-59y	6	3	6	3	18
60 + y	5	2	0	1	8
**Totals**	21	14	23	10	68

Interviews with herbalists and patients were semi-structured and wide-ranging. Researchers used standardised interview guides, arranged around key topics with associated prompts and probes. Patient interview topics included: contextual/background information; the relative importance of trust in decision-making; signal reading and interpretation; signal reliability and trustworthiness [Online file A]; previous experience and learning over time. Herbalist interview guides followed a similar format, but focussed more on signal *production* and perceptions of patient interpretation [Online file B]. Although, understandably, some patients were anxious not to be delayed for too long, most people were eager to share their experiences, generating a rich dataset. Additionally, twenty key informant interviews were conducted in the capital cities with individuals selected according to their relevance to the research topic: regulators, herbalists' associations, and relevant private/voluntary sectors organisations. Interviews were conducted in English or local languages, according to interviewees’ preferences.

Detailed hand-written notes were made during each interview and were typed up, along with observational field-notes, at the end of each day, with all materials translated into English for analysis. Two of the authors independently and manually coded all materials thematically. A series of *deductive codes* was created, based on the four ST-derived predictions; these included (*inter alia*) ‘real’ and perceived signal costs and pay-offs; knowledge/perception of signal reliability; and perceived relationships between signals and underlying ‘properties’. *Inductive coding* was performed simultaneously, based on the principles of Grounded Theory (Strauss and Corbin, 1998), whereby theoretical insights emerge from the data rather than being pre-specified. Inductive codes that did not derive *a priori* from Signalling Theory were determined through close reading and re-reading of transcripts.

Two caveats should be noted. First, because patients were contacted at herbal clinics, the sample excluded anyone who completely rejected this form of medicine. This approach is justifiable because our interest is in how patients choose *between* clinics, rather than why they utilise this particular sector. Second, because our 2015-16 study involved all herbal clinics identified in each city, this includes the three previously studied in Cape Coast; the sites where predictions were made and further investigated are therefore not fully independent.

Ethical approval was granted by Durham University's Anthropology Ethics Committee (UK); Oxford University's Social Sciences & Humanities Inter-Divisional Research Ethics Committee (UK); the Tanzanian National Institute for Medical Research (Lake Zone Institutional Review Board); and the University of Cape Coast Institutional Review Board (Ghana). All names have been changed and some other details modified to protect confidentiality.

## 3 Results and interpretation

### 3.1 Consulting a herbal clinic: risk and uncertainty

The patients we interviewed were diverse with regard to gender, age and socio-economic/educational background, although women and younger adults were in the majority: Table 1. They sought treatment for many different kinds of conditions, especially chronic complaints for which biomedical treatment is widely believed to be inadequate: infertility, ‘sexual weakness’, menstrual ‘irregularities’, haemorrhoids, chronic pain and fatigue, ‘diabetes’, ‘stroke’ and ‘pressure’ [hypertension]. Almost all had previously sought biomedical treatment; many had also tried other therapies (including other herbal clinics). Some were generally favourably disposed to herbal medicine and came full of hope; others arrived reluctantly as a last resort. Herbal clinics charged from US$2–3 up to $75 or more per visit – a significant financial outlay for many patients.

Given our sampling strategy, all interviewees believed (or hoped) that some herbalists were well-intentioned and able to give effective treatment. However, it was widely recognised that not all could be trusted. Some were just out to ‘*do business*’ as many interviewees put it, with little interest in patients' wellbeing. In Tanzania, fears were heightened by recent high-profile media exposés of fraudulent practitioners. Operating in this context of high uncertainty, the stakes for patients are high: choosing the wrong healer could result in continuing or even worsening ill-health, while many could ill-afford further unsuccessful treatment. There was also much at stake for the herbalists. A successful clinic could be very lucrative but, with intense competition and regulatory pressures, many struggled to make a decent living and going out of business was a real threat.

According to Signalling Theory, this combination of high uncertainty and intense competition should lead to prolific and differentiated signalling: the predictions to which we now turn.

### 3.2 Signals of trustworthiness (primary trust problem)

Patient interviews indicated the importance of finding a herbalist whom they could *trust* to be technically **competent** and to act with **integrity** (in the patient's best interests). As we discuss below, trust was not the *only* consideration for patients, especially those who were very financially constrained and/or desperate. For most, however, technical competence and integrity were important qualities in a healer, a finding congruent with other studies (Gilson, 2003, Gilson et al., 2005, Tibandebage and Mackintosh, 2005, Tarrant et al., 2010, Russell, 2005, Mechanic and Meyer, 2000, Gopichandran and Chetlapalli, 2013).

However, neither competence nor integrity is directly observable, so patients have instead to look for **signs** associated with these properties. According to the interview data, technical competence was indicated by: knowledgeability; a ‘modern’, ‘scientific’ approach; traditional healing ‘pedigree’; general popularity and track record; third-party validation; and the healer's confidence in his own abilities. Honesty, empathy, a caring manner and religious observance were seen to be indicative of integrity (Table 2). However, while these signs are somewhat less abstract than their underlying properties, they are also not easily observable and must therefore be communicated by more deliberately displayed and visible **signals**.

Our first prediction – that herbalists will use a range of signals to communicate their trustworthiness to patients – is clearly supported by the data. Table 2 lists the main signals identified in both settings, which operate across different distances as patients first make the decision to travel to a clinic, then to step inside, and to proceed with treatment. Some refer to the individual herbalist; others to the clinic (although in practice the two were often conflated by patients). Notably, despite some contextual differences, the signals displayed are remarkably similar in both countries.

**Table 2:** Signs and signals used by herbalists and clinics to convey properties of trustworthiness: competence and integrity.

Sign	Signal
Modern/scientific approach	• High-tech equipment/machines • Biomedical pictures/diagrams • ‘Hospital-like’ clinic appearance • Clean/‘hygienic’ setting • Well-packaged medicines, labelled with dosages, expiry dates, etc. • Computerised patient records • Business cards • Use of titles (‘Dr’, ‘phytotherapist’, etc.) • Referring patients for biomedical diagnostic tests • Smart attire, white clinical coat • Use of scientific/biomedical language in consultations, websites, *radio, etc.
Traditional healing pedigree/expertise	• ‘Traditional healer’ paraphernalia/dress • Display of roots, leaves, bark, etc. • Medicines in ‘traditional’ unlabelled containers • *Claims on websites and radio
Knowledgeability	• Sounding knowledgeable/convincing on *radio and in consultations.
Quality of medicine production/ingredients	• Medicines produced in ‘proper factory’ • Medicine is relatively expensive • ‘Natural’ ingredients
Popularity	• Large clinic with large waiting area • Busy/full clinic • Long-standing business • Widely-recognised brand • Medicines widely sold in other outlets.
Track record	• Personal experience • Testimony of known/trusted others • General reputation within and beyond local community • *Testimony from unknown others on radio, website, etc. • Long-standing business
Third-party validation	• Display of certificates (accreditation, qualification), etc. • Medicines bearing official (FDA) accreditation. • Referrals from biomedical clinicians. • Prominent on high-profile media
Virtue, honesty, putting patients' interests first	• Not charging ‘too much’. • Not over-claiming capabilities; referring patients elsewhere. • Church attendance • Praying with patients • Display of religious symbols • Virtuous lifestyle (no adultery, drunkenness, etc.)
Empathy	• Caring/empathetic manner with patients. • *Sounding caring/empathetic on radio. • Follow-up with patients post-treatment
Healer's self-belief	• *Sounding convincing and confident on *radio and face-to-face. • Giving medicine on credit (payment on results). • High financial investment.

[*Ghana only].

**Fig. 1:**
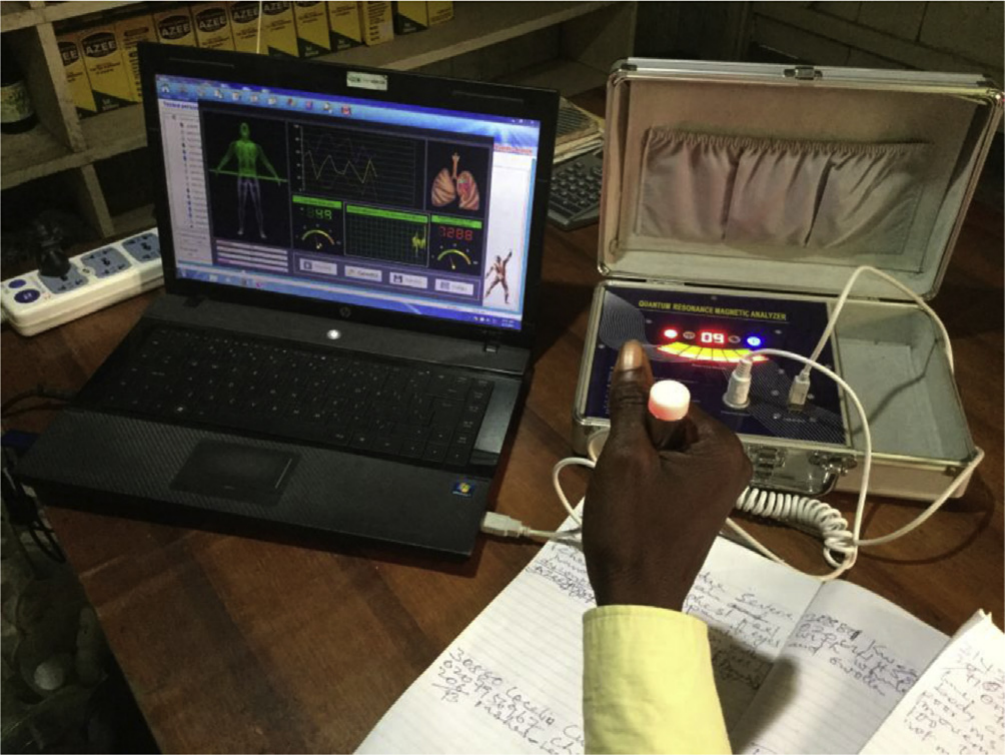
Quantum magnetic resonance analyser.

To take one example, SH's clinic in Cape Coast resembles a ‘modern’ biomedical facility. Patients enter a smart reception area where a receptionist measures their blood pressure, recording the details on a computer. After sitting in the waiting room, adorned with anatomical diagrams and shelves of medicine bottles (all FDA-approved and labelled with indications, contraindications, dosage and expiry dates), patients enter a consultation room to see the ‘doctor’, dressed in a white coat and wearing a stethoscope. Accreditation certificates hang on the walls, alongside pictures of Jesus and shelves with more medicine bottles (these ones unlabelled). At the back of the room, behind a curtain, is an examination couch. On a large desk sits a ‘*Quantum Magnetic Resonance Analyser*’: an impressive-looking (but not clinically-validated) device that purports to produce vast amounts of clinical information, from ‘cholesterol levels’ to ‘reproductive function’, based on a non-invasive procedure of holding a pulsating electronic probe linked to a computer [Fig. 1].

SH holds no medical qualification but is always addressed as ‘*doctor*’; he also uses the designation ‘*phytotherapist*’, while his website describes him as an ‘*African Traditional Healer, descendant of a long line of Botanists, Herbalists, and Traditional Healers for many generations*’. Like many Ghanaian herbalists, SH has a regular slot on several local radio stations, where he discusses various conditions, and responds to questions from the presenter and listeners, who also call in to testify to his skills.

There are lots of signals operating here, indicating expert knowledge, pedigree, accreditation from trusted third parties, religious observance, etc. Observational and interview data indicate that these signals – and those in other herbal clinics – are not there by chance; they are deliberately and strategically deployed to convince patients of the herbalist's trustworthiness in the face of intense competition. SH is currently investing in tiling and air-conditioning, “*so that people feel really good when they walk in*,” he explains. “*If they see me so neat and the environment clean, they will easily accept whatever I recommend to them*.” This was a common strategy in both countries; for example, DM (Tanzania) had set up his clinic to “*look like a modern hospital so patients are impressed*”. Patients were, indeed, impressed: several of DM's patients commented that his ‘*advanced clinic*’ resembled a hospital; an inference also made by patients in Ghana:

“*The place looks like a proper hospital: out-patients, pharmacy, lab, consulting room, nurses in proper attire. I am hoping that their medicine will be nice and heal fast like the clinic and services are nice*.” [48y woman, LK clinic]

SH also invests considerable effort in his radio performances, making careful preparations in advance: ‘*So when people hear me speak, they respect me because I know what I'm doing. […] they are all impressed.*” Likewise, KW (another Ghanaian herbalist) told us:

“*People have heard me speaking well on radio […] Questions are put to me and I answer them. The answers inform people about what you are doing, how you do it and it will ultimately attract them. […] When I explain things on air, the type of diseases that I cure, their symptoms, when they are convinced, they will come.*”

KW's patients are indeed convinced by this display of knowledge: “*When I heard him on the radio, and how he was explaining things and the assurance he gave out, I was convinced that he would be able to solve my problems*,” said one [45y woman], while another was persuaded by KW's empathic manner: “*The doctor was patient and explained things clearly, so I thought he would be patient and have time to listen*” [33y woman]. Patient testimonials from unknown callers were particularly compelling for many; one of NS's patients explained: “*On the radio, he spoke about so many sicknesses he could cure if you come to see him. […] Afterwards, they brought the phones lines out and people phoned in to testify to how he had healed them and that convinced me*.” [35y man].

Herbalists were adept at tailoring their signals to respond to patients' concerns and interpretations. For example, in both countries, there was widespread suspicion of healers who over-claimed their abilities. Such healers are thought to lack integrity, placing profit before patients' interests; as one interviewee in Tanzania explained, “*If a herbalist claims to be able to treat everything, it's a sign that he's a fake. A genuine herbalist will advise you to go to someone else if he can't heal you,*” [35y woman]. Knowing this, herbalists were careful to acknowledge the limits of their expertise, referring patients to hospital if there were unsure. Likewise, charging too much was widely associated with lack of integrity. ‘Genuine’ healers, it was argued, were not out to make big money, so “*many of the fake herbalists are those who demand a lot of money*,” as one Tanzanian patient [25y woman] claimed. On the other hand, patients also distrusted medicines that were ‘too cheap’. “*If you are selling this medicine at a low price I will be suspicious, it may be fraudulent,*” reasoned one Ghanaian patient [23y man]. One way out of this quandary was to charge relatively high prices for medicines but offer free diagnosis and consultation, a strategy that was well-received by many patients: “*Here, the diagnosis and advice is offered free, so they are not just after money*,” said a Tanzanian interviewee [30y woman].

Some herbalists adapted their signalling strategies according to the audience, treading a careful pathway between different aspects of their practice. SH, for example, is aware that for some of his patients – particularly older ones, who have grown up relying on ‘traditional’ herbalists (*dunsinyi*) and possession priests (*okomfo*) – the ‘spiritual’ aspects of herbal medicine are very important. When interacting with such patients, he conveys this ‘spiritual’ dimension through, for example, reciting certain incantations and/or blowing on the medicines to invoke the assistance of the local ‘gods’; he also dispenses the medicines in large, unlabelled plastic bottles “*which is what they think they should get from a* dunsinyi”, he explains. By contrast, younger, urban, well-educated patients tend to be sceptical of ‘spiritual’ connotations. Their trust is built on the fact that processed herbal medicines “*are clean, have dosage and expiry date, and are certified by the Food and Drugs Authority*,” as one young woman [23y] in SH's clinic put it. In such cases, SH emphasises the ‘modern’, ‘scientific’ aspects of his work, deliberately distancing himself from more ‘spiritual’ practices. This positioning is also important vis-à-vis the powerful Pentecostal and Charismatic churches, who vehemently oppose ‘indigenous’ medical practices that smack of idolatry (Meyer, 1998).

### 3.3 The trustworthiness of signals (secondary trust problem)

Having described the primary trust problem – how signals of trustworthiness are displayed by herbalists and ‘read’ by patients – we now turn to the trickier secondary trust problem: given the possibility of fakery, how can patients determine which signals to trust? According to ST, a signal's trustworthiness depends on the relative costs and pay-offs of its production/display for genuine versus dishonest signallers. Costs comprise monetary outlays plus time, effort, etc. They include both known, up-front costs (e.g. premises rental or business registration) and *potential* costs together with the *risk* of incurring them (e.g. the chances of being caught selling unregistered medicine and possible penalties: fines, reputational damage, etc.). Pay-offs are what a herbalist can expect to gain (i.e. money; also perhaps less tangible things like satisfaction), again with varying degrees of certainty.

In line with our second prediction, the signals described above vary substantially in their costs and pay-offs, with some herbalists deliberately investing in more discriminating signals to differentiate themselves from less trustworthy practitioners. Towards the ‘*pooling*’ end, we can identify several ‘cheap’ signals that are easily manufactured, reproduced and displayed by both honest and dishonest herbalists at minimal cost. They demand little time investment, no third-party validation, with little/no sanction for dishonest signalling. The title ‘Doctor’ (or ‘phytotherapist’) is easily adopted and rarely challenged. Wearing a white coat or adorning a website with ‘scientific terminology’ is not difficult; neither is producing labels for medicine bottles with putative dosages and expiry dates, praying with patients, or displaying anatomical diagrams and/or religious imagery.

More *discriminating* signals require greater investment of time and/or resources that would only be worthwhile if a herbalist could expect substantial returns through a successful business. The lack of guaranteed return and/or existence of sanctions for dishonest use might deter an incompetent or disingenuous healer from investing in displaying such signals. For example, EL, a Ghanaian herbalist, deliberately used expensive embossed packaging for his medicines, which could only be purchased in bulk (50,000 packages at a time, costing GHS 50,000 [$12,500]), reasoning that, “*Those that copy cannot afford to do this – they make cheap ones without embossment*.” Others spoke in similar ways about major infrastructural investment.

Other signals towards the discriminating end require validation from trusted third parties: informal ‘referrals’ from ‘hospital doctors’, personal recommendations from friends and relatives, or accreditation from highly-trusted authorities. This latter is behind SH's strategic display of an Ontario Council for Herbal Medicine membership certificate, acquired on an extended visit to Canada. Accreditation in Canada “*makes people to trust me*” and “*adds to my credibility*”, SH explained, because ‘Western countries’ are thought to be more rigorous in their regulation and less open to corruption than Ghana.

### 3.4 Interpreting signal reliability

Our third prediction, that patients will read and interpret signals according to their knowledge/beliefs about the costs of display, is also borne out by the data but with important caveats. Most patients were clearly making such inferences in ways predicted by ST. For example, costly infrastructure investment was widely interpreted as a trustworthy signal because such investment would only make sense for a herbalist confident enough in his abilities to expect a significant return. In Tanzania, a patient reasoned that, *“because they have invested a lot, their medicine is more likely to be good*,” [25y woman] while another in Ghana followed a similar logic: “*No fraudulent person who is only after making money will invest so much in providing a nice place only to dupe people by selling fake medicine.*” [37y man].

Patients made similar inferences about medicine packaging; one Tanzanian interviewee said, “*It is difficult [unlikely] to use a lot of money to package bad medicines well. Those that lack money to package properly may also skimp on production*,” [30y man], while another told us:

*“They have followed correct procedures for packaging medicines, like sealed bottles to prevent dust. […] Those who have followed correct procedures for packaging medicines are likely to have followed correct procedures for making them*.” [35y man]

A similar logic was used by some to reason that only genuine healers, confident in their abilities, would offer medicines on credit (usually in the form of a small up-front fee with the balance paid on results). As one Tanzanian patient reasoned, a herbalist “*wouldn't do that if he didn't know his medicines are good*,” [31y man].

Local reputation was widely regarded as a particularly reliable indicator of trustworthiness. In signalling terms, this is extremely difficult to fake: reputation takes a long time to build up but is quickly shattered if patients are dissatisfied. Many people therefore preferred to consult a local practitioner, whose reputation could more easily be assessed. As one Tanzanian patient put it, “*It is impossible for a herbalist to have good status in the community if he doesn't have good medicines*,” [45y woman].

In these examples, patients were assessing signalling costs/pay-offs with reasonable accuracy, but this was not always the case. Wearing a white coat, displaying certain images or calling oneself ‘doctor’ can be done cheaply and easily by anyone, yet some patients believed these to be reliable indicators of trustworthiness. One of KW's patients, for example, wrongly assumed that his use of the title ‘doctor’ “*means he has gone through some training*.” [51y man, Ghana].

To take another example, many interviewees in Ghana believed that only ‘genuine’ herbalists can sound convincing on the radio. One patient explained, “*It would be difficult – you cannot just talk about something you don't know. This is a medical issue – if you don't know, you can't give convincing explanations*,” [82y woman]. Many assume that radio stations vet herbalists, only allowing ‘genuine’ ones to broadcast, but this is not the case: local stations depend heavily on selling publicity ‘packages’ to herbalists, who pay upwards of GHS 400 [$80] per month for a regular slot. Listeners may be unaware that ‘live’ interviews are in fact highly scripted in advance, or that endorsements from presenters are an expected part of the package. A healer with a poor track record might not want to risk being disparaged live on air in a phone-in show but, in practice, denunciations never happen, and friends can be roped in to give positive testimonials.

Many patients also appeared to over-estimate the discriminatory value of Government accreditation; as one Ghanaian interviewee put it, “*I don't have the capacity to determine if a herbal medicine is good or not – it is only the FDA that can do so. Once they have certified that it can be sold to the public, that means it is good*” [37y man]. FDA certification in Ghana requires significant capital investment: herbalists estimated the cost of registering a single medicine to be around GHS 2500–3000 ($625–750), including on-site inspection, testing and certification fees and other ‘incidental costs’. However, beyond that, requirements are fairly minimal. Medicines are tested only for acute toxicity – not for efficacy as most patients believe. Moreover, as several herbalists noted, it would be relatively easy to send the FDA a ‘specially-prepared’ medicine for testing, after which manufacturing standards may slip. Others spoke darkly about ‘*prominent people in the system*’ who could, for a fee, ensure medicines passed the tests, while in Tanzania, we were told that practice certificates could easily be faked.

### 3.5 Informational asymmetries and their consequences

**Fig. 2:**
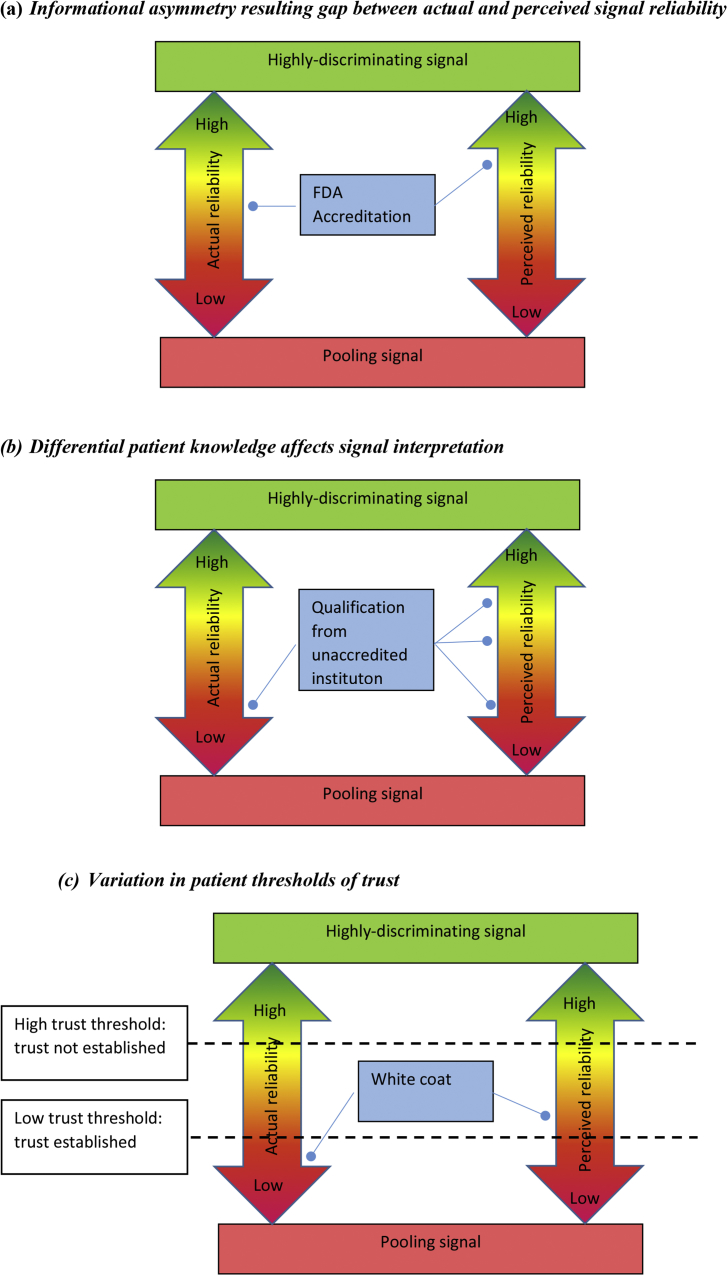
Comparing Actual [known by healer] and Perceived [by patient] Signal Reliability.

In each of these cases, the mismatch between *actual* and *perceived* signal reliability results from informational asymmetry. In order for patients to interpret signals ‘correctly’, they need accurate contextual knowledge. To assess the reliability of an accreditation certificate, a patient must know: how to read the certificate; who the accrediting agency is and how effective/reputable they are; what exactly is required for accreditation; how easy/difficult/expensive it would be to circumvent these processes (e.g. through bribery); how often/easily/cheaply certificates are faked; what the chances are of getting caught for non-compliance; what the consequences are of getting caught (financial, reputational, etc.); and the magnitude of pay-offs, (i.e. expected gains from operating a herbal clinic with/without official accreditation). In practice, many patients (especially older ones) are not literate, so cannot even read the certificate. Of those who can, very few are likely to have comprehensive understanding of the procedures involved, let alone be able to evaluate the costs, risks and pay-offs, as accurately as the herbalists, who have much more invested in this field. Patients typically therefore over-estimate the costs, and thus reliability, of this signal: Fig. 2a.

The level of informational asymmetry varies between patients according to their level of knowledge about a particular field (among other things). To take a different example, in Ghana, several institutions offer training programmes for herbalists; these range from a Bachelor's Degree in Herbal Medicine at the highly-respected Kwame Nkrumah University of Science and Technology (KNUST), to week-long courses on basic hygiene run by the Traditional Medicine Practice Council (TMPC), to a proliferation of online courses with varying degrees of accreditation and quality control. One Ghanaian Government regulator expressed concern about this situation, worrying that patients could easily misinterpret a TMPC short-course certificate as an indication of comprehensive training. Patients range from highly-educated professionals, well aware of the difference between a KNUST Degree and an unaccredited online course, to those for whom any ‘certificate’ looks impressive: Fig. 2b.

The *consequences* of informational asymmetry also vary between patients according to their personal circumstances and/or levels of desperation. A patient with few other options and/or who is desperate may accept a lower level of trust before proceeding with treatment than one in a less desperate or constrained situation. Meyer and Ward (2013) have argued that, when levels of risk and vulnerability are very high, trust loses its relevance in doctor-patient relationships that instead become characterised by *dependence* (see also Gilson, 2003). Many interviewees described long, arduous and often expensive therapeutic quests, finally coming to a herbal clinic desperate for relief, ready to try anything that might help. Such patients might be less discriminating, even perhaps deliberately ignoring signals that challenge or erode their hope. One (at KW's clinic in Ghana) said that a desperate patient doesn't care about appearances or expiry dates – all that matters is getting better. Likewise, those on minimal incomes cannot afford to be too choosy; they may have to accept a lower threshold of trust than someone in a more favourable position. This situation is illustrated in Fig. 2c, using the ‘white coat’ example. For a patient with a high trust threshold, the value of the ‘white coat signal’ is too low for trust to be established. However, someone with a lower trust threshold may accept the signal, despite its limitations.

A further, very important, consequence of the mismatch between the ‘real’ discriminatory power of a signal and patients' perceptions is that herbalists may not need to invest in the *most* discriminating signals in order to compete effectively in the market. If patients cannot effectively distinguish between mid- and higher-value signals, why take the time, trouble and expense of investing in the costliest ones? Certainly, none of the herbalists with whom we worked seriously contemplated taking the KNUST Degree because they didn't need to: patients came to them anyway and, as a signal of competence, an impressive-looking ‘machine’ was just as effective and much cheaper (in time and money) than a degree certificate. The upshot of this is a relatively undifferentiated mid-range herbalist medicine market, whereby the least trustworthy herbalists (who can only afford to display signals that are widely recognised as pooling) are weeded out but those who pass this threshold can remain competitive without over-investing in unnecessarily costly signals. In other words, herbalists have only to invest in signals that are costly *enough* to convince prospective patients to trust them.

### 3.6 Signal evolution

Signalling ‘games’ have important temporal dimensions, since both signallers and receivers engage in multiple interactions over time, enabling them to develop their knowledge and hone their strategies. However, unlike some other signalling situations, where the outcome is ultimately clear to both parties (see, for example, Gambetta and Hamill's (2005) work on taxi drivers in Belfast and New York who either were or were not robbed/killed by their passengers), with healthcare, it is much more difficult to know unambiguously – even after the event – whether the practitioner or medicine *was*, in fact, trustworthy. It is notoriously difficult to establish clear causal relationships between treatment and clinical outcome, making it difficult for patients to accumulate accurate knowledge about signal reliability. Powerful positive and negative feedback loops between trust and clinical outcome operate via the placebo and nocebo effects (Arnold et al., 2014). Moreover, many conditions resolve themselves regardless of (or despite) medical intervention, and the widespread practice of polypharmacy (attempting several treatments simultaneously) makes it difficult to distinguish the effect of each, while widespread beliefs that divine intervention plays a strong part in healing further complicate the business of ascertaining which herbalists – and therefore what kinds of signals – were in fact trustworthy.

Patients’ ability to read and interpret signals accurately is also affected by temporal shifts in the wider field. As stated in our final prediction, certain signals may lose their discriminatory power over time, as they become easier/cheaper to display and more widely adopted, and new signals of trustworthiness will emerge. Keeping abreast of these developments can be a significant challenge for patients who, unlike the practitioners, usually only engage infrequently in this field and do not have the same long-term incentives to update their knowledge regularly.

Take the example of herbalists' radio broadcasts, which have become increasingly popular over recent years. In the early days, when this was a novel forum, it may not have been clear that the chances of being ‘exposed’ by presenters asking tricky questions or callers complaining about poor treatment would be minimal. Thus, perhaps, only herbalists sufficiently confident in their knowledge and track record would be willing to take the risk of doing a live phone-in show. Now, herbalists know that the risks are negligible, providing they have the money to pay for airtime and the confidence to sound convincing. A good radio broadcast can now be achieved by both competent, empathetic herbalists and by confident, quick-thinking charlatans, while patients struggle to distinguish between the two.

A relatively new signal to emerge is the Quantum Magnetic Resonance Analyser (QMRA). Back in 2009, these machines were unknown; now they are on display in most up-market herbal clinics in both countries and have become a potent signal of trustworthiness for patients who don't distinguish them from the clinically-validated diagnostic technology encountered in hospitals. As one Tanzanian patient explained, “*even when you go to hospital you find doctors using machines to make diagnosis*” [32y woman]. Herbalists have been quick to capitalise on this development; as KW [Ghana] put it, “*I have to move with the times […] People believe the machines more than if you don't have machines*.” However, while widely regarded by patients as a highly-discriminating signal, in fact, QMRA machines are not clinically validated; they are relatively cheap (retailing for around US$200) and easy to use, and are not subject to any regulatory scrutiny. As these machines become more and more widely adopted, we would expect their perceived reliability as an indicator of trustworthiness to diminish and for other signals to emerge. As long as the market remains competitive and uncertainty remains high, it will continue to make sense for high-quality herbalists to invest in signals that are discriminating *enough* to communicate their trustworthiness to patients.

## 4 Discussion

We set out both to address an empirical question – how patients in Ghana and Tanzania come to trust/distrust herbalists – and to assess the wider theoretical contribution that Signalling Theory might make to the study of health-related trust problems. In relation to the former, our findings echo those from other studies: patients were seeking technically-competent, well-intentioned healers, and were actively reading and interpreting signals that they believe to be associated with those ‘properties’. While there were some differences between Ghana and Tanzania in the nature and degree of regulation, and in some of the strategies adopted by herbalists (for example, the use of radio broadcasts in Ghana), there was a remarkable degree of similarity observed between the two countries.

However, Signalling Theory has enabled us to go beyond *description* to formulate theoretically-derived *predictions* that are amenable – potentially – to empirical testing. Moreover, it moves us beyond the *primary* trust problem addressed by other researchers – cataloguing observable indicators of trustworthiness in healthcare providers – and provides tools for tackling the trickier *secondary* trust problem, where the trustworthiness of those indicators must be ascertained. Signalling Theory also enables a basis for comparative work between very different empirical contexts that share the underlying conditions of *uncertainty*, since we can hypothesise that what appear to be very locally-specific practices might in fact share a common underlying *logic.*

In practical terms, by giving us a framework for comparing the *actual* and *perceived* reliability of signals, ST might enable more effective targeting of interventions. To pursue the example illustrated in Fig. 2a, the gap between the actual and perceived value of official accreditation might be addressed in several ways. Carefully-targeted information campaigns could help raise public awareness of what accreditation actually entails, thereby lowering the *perceived* reliability of the signal. Meanwhile, improving the rigour of testing and increasing/enforcing penalties for non-compliance could help raise the signal's *real* costs and reliability. And of course, improvements in access to decent, affordable healthcare are likely to push up patient thresholds of trust (Fig. 2c), thereby narrowing possible windows for discrepancy.

Diminishing the gap between actual and perceived signal reliability should result in a dual benefit: first, by enabling individual patients to make more accurate assessments of herbalists' quality and, second, by incentivising herbalists to raise their game. As we have suggested above, as long as patients cannot accurately distinguish between some mid- and higher-range signals, it will not make sense (at least in ‘rational choice’ terms) for herbalists to improve their skills and competence by, for example, embarking on high-quality training programmes. If patients become more adept at reading and interpreting signals accurately, the herbalist market is likely to become more clearly differentiated, with demonstrably trustworthy/effective herbalists rising to the top rather than being indistinguishable – as is currently the case – from other, more mediocre, practitioners.

Of course, ST and other ‘rational choice’ approaches can only take us so far. Moreover, trust is clearly not the *only* factor shaping healer-patient interactions and attendant decision-making. Resource and infrastructural limitations continue to constrain health-seeking possibilities for large sectors of the population in Ghana and Tanzania, and treatment decisions relate also to convenience, ideology and personal preference. Medicines have affective and emotional meanings beyond just their instrumental value (Whyte et al., 2003), and the same has been shown to apply to trust in healthcare practitioners/institutions (Gilson, 2003, Ackatia-Armah et al., 2016, Meyer and Ward, 2013). In a recent edited volume, Broch-Due and Ystanes (2016) have argued persuasively that the ethnocentric assumptions of Western scholars have resulted in the privileging of individualistic, calculative models of trust, obscuring other modalities.

We are broadly in agreement with these arguments. However, expanding the ways that we think about trust should not preclude in-depth analysis of particular facets. We would not wish to suggest that all the complexity and nuance of patients-herbalist interactions can be reduced to a series of signal-based transactions. However, it is clear from our data that herbalists and patients *are* engaging signalling practices and that ST gives us some theoretical purchase in understanding these.

## 5 Conclusion

Our work suggests that Signalling Theory might usefully be applied to help understand, make predictions about, and perhaps begin to address, trust problems in healthcare, especially in LMICs where levels of uncertainty are high and formal regulation weak. To achieve this, we identify three key areas for theoretical and methodological development.

First, we need to work with different kinds of data and models, challenging and traversing standard disciplinary boundaries. We argue that much can be gained from combining the rigorous theoretical models offered by Game Theory with the nuance and contextual detail enabled by ethnographic enquiry. While this paper has focussed on ethnographic detail, an important next step could be to collect quantitative data that would enable more precise plotting of signalling mismatches illustrated in Fig. 2. Some relevant data could be obtained relatively straightforwardly: for example, average daily clinic takings or the official costs of registering a medicine (although not necessarily the ‘unofficial’ costs). However, other data present far greater challenges: where regulatory capacity is weak, record-keeping is often incomplete or non-existent. We found it nigh-on impossible to obtain reliable data on failure rates of medicine registration, for example, or the penalties incurred in a given time period for those failing to comply with requirements. In addition to these practical challenges, bringing together different kinds of data into a single analytical framework presents epistemological challenges, which will require ethnographers and formal Game Theorists to develop new collaborative ways of approaching problems.

This collaborative approach will be needed to address the new theoretical complexities that arise when ST is applied to health-related trust problems. For example, ST has generally assumed that trusting and trustworthiness can be treated as independent variables: this is clearly not the case with medicines where powerful feedback loops operate via the placebo and nocebo effects, respectively bolstering or undermining initial trust positions.

Finally, the herbalist-patient interactions described here are just one part of a complex, multi-layered set of health-related trust problems that pertain in uncertain and resource-constrained contexts, where actors are obliged to assess not only the trustworthiness of the individual/institution they are dealing with directly but also that individual's/institution's capacity and willingness to ensure trustworthiness further up supply chains. To truly apprehend the parameters of these ‘referred trust games’ played out at different scales is a major undertaking indeed.
