# Supplementary material for: The application of Signalling Theory to health-related trust problems: The example of herbal clinics in Ghana and Tanzania
Source: Soc Sci Med. 2017 Sep;188:109–18. doi: 10.1016/j.socscimed.2017.07.009 (PMC5559643; doi:10.1016/j.socscimed.2017.07.009)
Supplement: Online data [file mmc1.docx]

**Trust, risk and uncertainty in medicinal transactions in Sub-Saharan Africa**

**NOTES ON CONDUCTING INTERVIEWS WITH HERBAL CLINIC PATIENTS**

**February 2016**

**This is an interview guide, not a list of questions. It is intended to support what we covered in the training and remind you what we need to know about the ways that patients read and interpret signals of trustworthiness when visiting a herbal clinic and how these shape the transaction. Please read carefully.**

1. **Observation of the patient**

***Aim:* to understand enough about the patient’s background in order to provide context for trust decisions.**

*Observe/record:* gender, approx. age and appearance; alone or with someone else, etc.

*Ask:* Reason for visit.

1. **Decision-making around choice of herbal clinic: the role of trust, signals and constraint**

***Aim:* to establish the range of factors that influence a patient’s choice of herbal clinic and the role of trust in relation to other factors/constraints (price, distance, etc.).**

***Approach:* To find out *w*hy they chose to come to this particular herbal clinic at this particular time and what factors/experiences influenced this decision.**

*Suggested areas to probe:*

- Is this their first visit or have they been on previous occasions [how many? Over what time span? For (many) different conditions?]
- What considerations did the interviewee take into account in deciding to come to this particular clinic? **N.B. Look out for signals such as the following and probe but *do not prompt by asking leading questions* **
  - ‘Trust’ / quality factors may include: reputation, previous good experience, formal accreditation, quality of medicines, cost of medicines, range of medicines, personal manner and/or appearance of the herbalist, their knowledgeability/expertise, advertising, branding, etc.
  - ‘Constraint’/other factors may include: distance, ease of access, cost, waiting times, etc.
- What is the relative importance of these different factors?
- Does the patient use other healthcare providers (of a similar kind or different)? How do these compare and what influences the choice of which to go to?
- How does the interviewee rate the quality and/or trustworthiness of the medicines that s/he obtains from this clinic? [Overall? Compared to other herbal clinics? Compared to other kinds of healthcare providers?]

1. **Signals of trustworthiness:**

***Aim*: To establish the signals used by the patient to establish trustworthiness (of the herbalist, clinic and medicine), how these are valued and how adept they are at ‘reading’ them accurately.**

***Approach*: To ask further probing questions (why, how, what…) on each of “trust” and “constraint” factors identified above.**

***The questions will depend on which “trust” and “constraint” factors the interviewee considers to be important in their decision to come to this particular clinic, for example:***

- **Price:** Why is price important? What can/does price indicate to you? Is price indicative of drug quality / reliability? Etc.
- **Knowledgeability/expertise of herbalist**: How did you come to know that this person is knowledgeable? [Personal experience? Reputation? Advertisement? Radio broadcast, etc.?] How can you tell they are knowledgeable? What kind of expertise is important to you? [e.g. wide knowledge of medicines, in-depth expertise on a particular disorder/medicines, etc.?] How do you value different *sources* of knowledge [e.g. from formal training programmes, from long experience, being taught informally by someone…] Is it always important to obtain medicines from someone who is knowledgeable or does it depend on what you need [e.g. if you know the medicine/condition well, may be less important?]

*****Continue to ask similar questions for every signal identified.***

1. **Reliability of signals**

***Aim:* For each factor/signal, to establish how easy/costly it would be for an untrustworthy herbalist [i.e. dishonest and/or incompetent] to fake, and what the consequences might be if they were ‘caught’.**

***Approach*: To ask further probing question on each signal identified by the patient**

***The questions will of course depend on which signals are being used. For example:***

- **Knowledgeability:** How easy is it to be sure that the information you are being given by the herbalist is accurate? How easy is it to tell which herbalists are genuinely knowledgeable and which are not? How easy might it be for someone to appear to be knowledgeable when they are not really? Do such people ever get found out? If so, how often does it happen and what are the consequences for them? And what are the consequences for the patient of going to an unknowledgeable herbalist?
- **Certificate of practice:** Do you know how the certificate is obtained and what the herbalist has to do to get it (and retain it)? [E.g. financial cost, training requirements, quality control, etc.] How good an indicator of quality is having a certificate? Could someone who isn’t properly qualified/registered get hold of such a certificate [e.g. faking or buying on black market?] Ever heard of anyone getting caught doing this? If so, how often and what were the consequences?

****Continue to ask similar questions for every signal identified.**

1. **Bad experiences [i.e. what happens when the signals are misread or not acted on?]**

***Aim*: To identify difficulties in reading and interpreting signals, which have led to ‘bad’ trust decisions on the part of patients.**

***Approach:* To ask about negative experiences and times when signals might have been misread or misinterpreted.**

***Suggested areas to probe***

- Have you ever had a **bad experience** with herbal clinics/medicines (not working, harming you, etc.)? Explain exactly what happened in as much detail as possible, including where/how/why you got the medicine, and the consequences.
- Is it possible, in hindsight, to identify signals that might have alerted you to the possibility that this herbalist/clinic/medicine would not be a good one?
- Do you know [or have you heard of] **anyone else** who has had a bad experience with herbal clinics/medicines? Again, get the full story in as much detail as possible.

1. **Concerns about fakery and mimicry / other challenges**

***Aim*: To understand the baseline context and levels of uncertainty, as experienced and understood by the patient. This is the basis against which trust must be produced.**

**Approach: to ask specifically about the possibility of fakery/mimicry and how likely this is to happen.**

***Suggested areas to probe***

- Do you ever think about the possibility that some herbalists may not be honest or may not know enough about the medicines?
- Try to distinguish between herbalists who are:

1. Fraudulent / dishonest
2. Just incompetent and don’t know enough. [N.B. Some may be both.]

- If so:
  - How big/widespread a problem is this? (i.e. how common are bad/incompetent herbalists and what are the implications?)
  - Do you take any steps to try to ensure that you consult herbalists that are good? Are there any particular challenges you face in doing this?

1. **Any further comments?**
2. **Thank the respondent and end the interview.**
