# Supplementary material for: The application of Signalling Theory to health-related trust problems: The example of herbal clinics in Ghana and Tanzania
Source: Soc Sci Med. 2017 Sep;188:109–18. doi: 10.1016/j.socscimed.2017.07.009 (PMC5559643; doi:10.1016/j.socscimed.2017.07.009)
Supplement: Online data [file mmc2.docx]

**Trust, risk and uncertainty in medicinal transactions in Sub-Saharan Africa**

**NOTES ON CONDUCTING INTERVIEWS WITH HERBAL CLINIC PRACTITIONERS**

**February 2016**

**This is an interview guide, not a list of questions. It is intended to support what we covered in the training and remind you what we need to know about the ways that herbalists produce signals of trustworthiness, how they respond to the way that patients are likely to ‘read’ these signals, and how these shape the transaction. Please read carefully.**

1. **Context:**

***Aim:* to describe and understand the physical context of the herbal clinic.**

***Approach*: Observation of the herbal clinic, its situation, and the herbalist and other staff.**

*Some things to look for:*

- Provide detailed observational notes on the location and set-up of the clinic, including waiting room, consultation room, facilities available, etc.
- What is the *range* of herbal products/therapies available?
- Establish broad parameters of the patient base [i.e. local/distant, socio-economic status, major conditions presenting].
- Background on the herbalist (and, where relevant, other clinic staff): observe gender and approximate age; ask about the history of the clinic and its current operation.

1. **Establishing the range of signals produced**

***Aim*: To establish the range of signals being used by the herbalist to convey trustworthiness.** *By ‘signals’ we mean ways that the herbalist might try to communicate to prospective patients that they and their products are trustworthy.*

**Approach: observe and ask.**

- N.B. Some signals might be directly observable [e.g. smart appearance of clinic/herbalist, personal manner, certificates of practice, wide range of products in view, etc.]. These should be noted down.
- Ask what it is that attracts patients to this particular herbal clinic, and prompt/probe where necessary to cover possibilities such as : reputation/word of mouth, price, quality of service (which aspects?), personal manner, range of medicines available, *quality* of medicines available, professional certification/registration, any advertising, etc. BUT *do not prompt by asking leading questions* **

1. **Establishing the cost and importance of signals**

***Aim*: For each signal, to establish the cost and importance of that signal for both trustworthy [honest, competent] and untrustworthy [dishonest and/or incompetent] herbalists.**

***Approach*: Go through each of the signals identified above, seeking as much detail as possible on costs and importance.**

***The exact questions will depend on the signals that you have identified; here are some examples:***

- Certificate of registration/practice: What procedures are required to obtain a certificate? How much money? Any training requirements? Any test/examination? Any ongoing quality control? How easy would it be to fake a certificate, what would be the implications of getting caught? How often are people caught faking certificates?
- Knowledgeable response to patients’ questions: How did the interviewee gain this knowledge? Participation in formal training programmes? Length of experience/service? How easy would it be for someone who wasn’t well informed to appear knowledgeable and dupe patients? What would happen if such a person were ‘caught’? How often does this happen?
- Reputation: How long did it take to build a reputation? What difficulties were experienced during this process? How did you manage before you had secured the reputation? What happens to others starting up without a reputation? How easy is it for a good reputation to be broken? What happens?

1. **Establish the decision-making processes behind the adoption of certain signals**

**Aim: To establish why this herbalist decided to invest in particular signals and not others. Also to ascertain whether the herbalist is differentiating between patients in signalling strategies.**

***Approach*: Go through each of the signals identified above, asking further ‘why’ questions.**

***Again, the questions asked will depend on which signals that are being used, but for example:***

- Why did the herbalist elect to take a particular training programme, or choose to display certificates / licences, etc.?]
- Try to establish whether ***particular signals*** are aimed at ***particular types of patient***. [e.g. more/less educated, young/old, etc.].

1. **Experiences of ‘bad’ / ‘dishonest’ herbalists:**

***Aims:* To understand the degree of uncertainty about herbalist ‘quality’ and to differentiate between the signalling practices of ‘good’ and ‘bad’ herbalists.**

***Approach*: To establish how widespread the phenomenon of bad/incompetent herbalists is in the interviewee’s experience and to understand how the signalling practices of ‘untrustworthy’ (incompetent and/or dishonest) practitioners contrast with those of trustworthy ones: i.e. whether they are using different signals, how easy/difficult it is to distinguish, and what the consequences might be of dishonest/poor practice.**

***Some suggested probes:***

- Are you aware of any herbal clinics/practitioners whose medicines/practice are dubious or of low quality? *[N.B. The respondent does not need to name them.]*
- If so, how widespread a problem is this?
- Describe the practices of these untrustworthy herbalists. What kinds of practices do they deploy and what is ‘wrong’ with them? Are they dishonest [i.e. deliberately selling poor-quality/fake medicines] or (just) incompetent [ie. Not sufficiently knowledgeable, skilled, or experienced]?
- How easy is it **for you** to distinguish a ‘good’ herbalist from a ‘bad’ one? How would you distinguish?
- How easy is it **for a patient** to distinguish a ‘good’ herbalist from a ‘bad’ one? How could they distinguish?
- What (if anything) happens to ‘bad’ herbalists? Do they get found out? What are the consequences for them and their business? Any examples? Get as much detail as possible.
- Does the existence of ‘bad’ herbalists affect your business in any way? How? What steps (if any) do you take to overcome this?

1. **Any further comments?**
2. **Thank the respondent and end the interview.**
